# Overview of the Role of Spatial Factors in Indoor SARS-CoV-2 Transmission: A Space-Based Framework for Assessing the Multi-Route Infection Risk

**DOI:** 10.3390/ijerph191711007

**Published:** 2022-09-02

**Authors:** Qi Zhen, Anxiao Zhang, Qiong Huang, Jing Li, Yiming Du, Qi Zhang

**Affiliations:** 1 School of Architecture, Tianjin University, Tianjin 300072, China; 2 Department of Epidemiology and Biostatistics, School of Public Health, Tianjin Medical University, Tianjin 300072, China

**Keywords:** spatial factors, indoor SARS-CoV-2 transmission, infection risk assessment, multi-route transmission, occupant behavior, COVID-19

## Abstract

The COVID-19 pandemic has lasted from 2019 to 2022, severely disrupting human health and daily life. The combined effects of spatial, environmental, and behavioral factors on indoor COVID-19 spread and their interactions are usually ignored. Especially, there is a lack of discussion on the role of spatial factors in reducing the risk of virus transmission in complex and diverse indoor environments. This paper endeavours to summarize the spatial factors and their effects involved in indoor virus transmission. The process of release, transport, and intake of SARS-CoV-2 was reviewed, and six transmission routes according to spatial distance and exposure way were classified. The triangular relationship between spatial, environmental and occupant behavioral parameters during virus transmission was discussed. The detailed effects of spatial parameters on droplet-based, surface-based and air-based transmission processes and virus viability were summarized. We found that spatial layout, public-facility design and openings have a significant indirect impact on the indoor virus distribution and transmission by affecting occupant behavior, indoor airflow field and virus stability. We proposed a space-based indoor multi-route infection risk assessment framework, in which the 3D building model containing detailed spatial information, occupant behavior model, virus-spread model and infection-risk calculation model are linked together. It is also applicable to other, similar, respiratory infectious diseases such as SARS, influenza, etc. This study contributes to developing building-level, infection-risk assessment models, which could help building practitioners make better decisions to improve the building’s epidemic-resistance performance.

## 1. Introduction

As of 29 April 2022, the total number of COVID-19 cases globally exceeded 566,977,818, with more than 6,376,503 reported deaths [1]. Despite 12,219,375,500 vaccine doses, SARS-CoV-2 variants such as Delta and Omicron are highly contagious and may evade vaccination and the immune defenses of infected individuals. The basic reproduction number R0 of the coronavirus, representing the average number of people infected by an infected person, ranges from 1.90 to 6.49 [2]. It remains important to understand how the virus is transmitted, accurately assess the infection risk, and implement appropriate nonpharmacological interventions. Cases have been reported in various public places including hospitals, markets, churches, bars, restaurants, offices, and schools in the past two years [3]. With evidence accumulating during the COVID-19 outbreak, the World Health Organization (WHO) points out that most transmission occurs between people in close contact and that inhalation of aerosols or touching the facial membranes with contaminated hands may also cause infection [4].

While aerosol transmission of COVID-19 is not listed as the main route of transmission, 239 scientists have published an open letter to the WHO supporting and proving that aerosol transmission was the most likely mechanism explaining the spatial pattern of COVID-19 infections [5]. Virus transmission in the outbreak of COVID-19 in an air-conditioned restaurant in Guangzhou cannot be explained by droplet transmission alone. Virus-laden small (<5 μm) aerosolized droplets can remain in the air and travel long distances, which leads to the infection of distant customers [6]. In addition, evidence for aerosol transmission involving church [7], cruise [8] and buses [9], etc., have also been reported. In order to control the pandemic, all potentially important transmission routes must be interrupted, including providing sufficient and effective ventilation, high-efficiency air filtration, and avoiding overcrowding, etc. Reported outbreaks of COVID-19 occurred primarily in indoor settings [3]. Most of the research on the influencing factors of indoor infection risk focuses on environmental and behavioral aspects, such as ventilation, wearing masks, and surface disinfection. Poor ventilation increases the infection probability, and many studies have been reported on the relationship between ventilation strategies [10], ventilation rates [11], and air organization [12] with infection risk. In addition, air temperature (AT) and relative humidity (RH) and ultraviolet (UV) light have significant effects on virus viability [13]. Wearing a mask can help filter most virus-containing droplets and aerosols, and handwashing can effectively reduce the risk of infection by touching fomite [14]. Aspects of close contact, such as posture [15], handshake [15], coughing [16], etc., also affect the pattern and level of exposure to the virus.

Architectural design has a significant impact on indoor natural ventilation, occupants’ movement, behavior, interactions with the environment interface and each other, and the spread of microorganisms [17]. Shao et al. [12] emphasized the obvious spatial heterogeneity of virus distribution. Proper spatial orientation and openings provide adequate natural ventilation to reduce the accumulation of infectious aerosols. Furthermore, spatial features have proven to have predictable effects on occupant movements [18]. Compared to enclosed-plan offices, employees in open-plan offices spend less time sitting down and have more face-to-face interactions [19]. The epidemic-prevention performance of buildings has not received enough attention, and few researchers have specifically explored the role of spatial factors in indoor risk assessment. 

Some risk assessment models have been proposed to help reduce the risk of indoor transmission. Peng et al. [20] presented a tool to calculate the risk of airborne infection in some basic scenarios. Based on this, Laborda et al. [21] developed the aerosol risk assessment website, which clearly presents the input parameters and their results. Although multiple routes of transmission of respiratory infectious diseases are undeniable, to date, only a few studies have explored the comprehensive risk in a space formed by the superposition of multiple routes. Azimi et al. [8] established a modeling framework of virus spread on the Diamond Princess cruise ship to evaluate the relative importance of three transmission routes. The Harvard Health Building team released an online calculator [22] and came up with prevention guidelines for different buildings [23]. Gao et al. [24] developed a mathematical model to study the relative contributions of different transmission routes in enclosed spaces based on a location access network and introduced three dose-response coefficients associated with influenza viruses. Duives et al. [25] developed a combined model, linking microscopic pedestrian movement and virus transmission that can be used to describe any specific individual movement and behavior. In this model, the indoor airflow diffusion is simplified as uniform and cannot reflect the effect of ventilation mode and spatial arrangement. The differences in indoor infection risk caused by changes in spatial parameters are difficult to quantify due to the lack of suitable methods and tools.

This study aims to provide insights into the spatial factors involved and their roles in the indoor spread of the virus and to propose a spatial-based, multi-pathway infection-risk assessment framework. Section 2 reviews the transport process of the virus from infected to susceptible and the classification of transmission routes. Section 3 summarizes the spatial factors involved in virus transmission and how they affect virus spread and survival. A comprehensive infection-risk assessment framework containing complex interactions of indoor spatial information, occupant behavior information, and virus transmission information is proposed in Section 4. The occupant behavioral parameters involved in the framework are also summarized.

## 2. Methodology and General Result

To analyse different aspects of this topic, we conducted a broad search on the databases “Web of Science” and “Google Scholar”. The search string is a combination of different keywords: (“built environment” or “indoor environment” or “indoor space” or “spatial design” or “spatial factor” or “building design” or “architectural design” or “design factor” or “room”“) and (“COVID-19” or “COVID” or “SARS-CoV-2” or “coronavirus” or “epidemic” or “pandemic” or “respiratory disease” or “infectious diseases”) and (“relationship” or “association” or “effect” or “impact” or “influence” or “connection” or “correlation” or “link” or “linkage”). 

**Figure 1:**
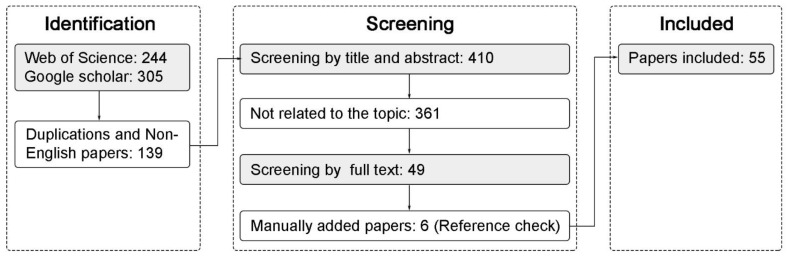
Diagram of literature selection process.

Initially, the broad search returned 549 papers, 139 of which were duplications or not in English. Through the screening of the titles and abstracts, 361 weakly relevant papers were found and eliminated. The main inclusion criteria in this stage were those papers that have at least one spatial design factors affecting the spread of pathogen. The full texts of 49 papers were reviewed and 32 papers were added manually from the reference checks. Finally, 55 papers were relevant to our main review questions and were included in our analysis (Figure 1).

## 3. Multi-Route Transmission of SARS-CoV-2

### 3.1. The Emission of Infectious Respiratory Fluids

SARS-CoV-2 is the virus that causes COVID-19, and its genetic material is packaged in an outer layer (envelope) of proteins and lipids. The virions are spherical in outline, with diameters ranging from 60 to 140 nm [26]. SARS-CoV-2 is transmitted by exposure to infectious respiratory fluids, which are generated in the human respiratory system, and is the main carriers of respiratory infectious pathogens. Respiratory fluids can be produced in four parts of the respiratory tract, namely lungs [27], trachea [28], larynx [29] (close and open the vocal cords), and oral/nasal passages [30] (close and open the mouth/breath). People release respiratory fluids in the form of droplets across a spectrum of sizes along with the airflow during exhalation (breathing, speaking, singing, coughing, and sneezing). Severe expiratory events (coughing and sneezing) produce more large and high-speed droplets that are easily visible to the naked eye [31]. Large droplets are generated near the front of the oral cavity and small droplets are generated by the vocal folds during vocalization. Therefore, speaking and singing emit larger droplets on average than breathing, and the emission rate increases with volume [32,33].

The respiratory droplets (RDs) and expiratory flow features involved in expiratory activities determine the fate of droplets, including evaporation, deposition, aerosolization and exposure patterns. Previous studies have investigated the production and characteristics of exhaled droplets by experiment, including solid impaction, high-speed photography, optical technology, charge separation, etc. [34]. Table 1 shows the characteristics of droplets involved in breathing, speaking, coughing, and sneezing including size range, emission rate, particle concentration and velocity. Due to the individual differences of subjects and differences in testing methods and tools, the data obtained from the survey are obviously inconsistent. The diameter of RDs produced by breathing is generally less than 100 μm [34], while the size range of RDs produced by speaking, coughing and sneezing has no significant difference [16], which is less than 2000 μm [35]. In general, the more intense the expiratory activity, the higher the particle concentration [16,33,35,36,37] and emission velocity [16,38,39,40,41,42,43,44,45], and the higher the RDs emission rate [16,32,35,46]. In addition, there are individual differences in the direction and diffusion angle of RDs [47].

### 3.2. Evaporation, Deposition and Dispersion

**Figure 2:**
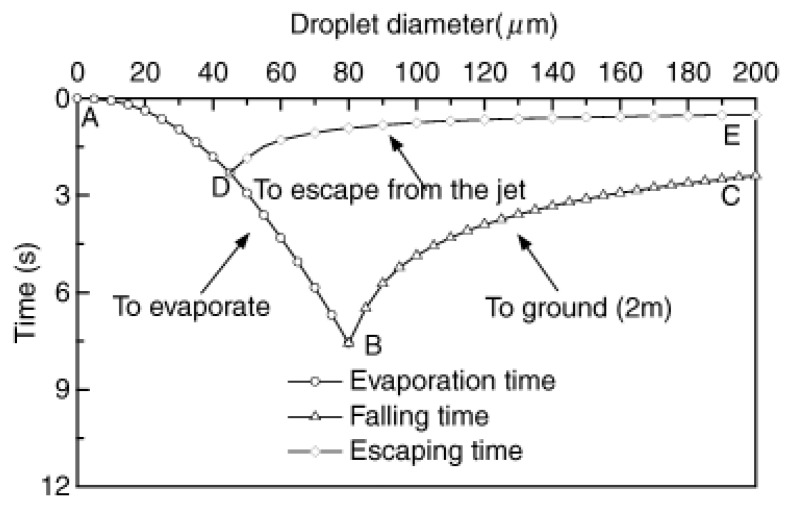
Evaporation, falling and escape time of RDs of varying diameter (T = 20 °C, RH = 50%, V = 10 m/s) [50].

After being expelled, RDs are exposed to relatively dry air as they move through the ambient environment, and their initial size determines their evaporation, deposition, and dispersion. Large droplets >100 μm will fall to any surface encountered (including room surfaces, body parts and clothing) under the force of gravity following a ballistic trajectory (typically land within 1–2 m from the source). Most particles ≤50 μm evaporate into dry particles or aerosols during falling, which is suspended in the air long enough to be inhaled by susceptible people over short or long distances [48]. The droplet trajectory is mainly affected by gravity, inertia and aerodynamic resistance, and the detailed dynamic process can be understood through numerical models. The classic Wells evaporation decline curve [49] first revealed the relationship between size, evaporation and fall time of RDs. Xie et al. [50] proposed an optimized physical model for the evaporation and movement of RDs and investigated the evaporation, falling, and escape time of RDs with different diameters (Figure 2). The intersection B represents the maximum droplet diameter that fully evaporates before descending 2 m, while the intersection D is the maximum droplet diameter that fully evaporates in the exhaled jet. However, these assume that the AT and RH in the space are uniform and there is no surrounding airflow interference.

There are also many factors that affect droplet evaporation and deposition rates, such as initial droplet velocity, temperature, humidity, turbulence, etc. Elevated ambient temperature can accelerate the process of droplet evaporation. The median evaporation time for droplets with a diameter of 12 μm decreased by 71% (from 1.44 s to 0.42 s) as the ambient temperature increased from 5 °C to 35 °C [51]. Dry air accelerates the droplet evaporation process, with critical dimensions ranging from 60 μm to 125 μm at 0% to 90% RH. Recent studies [52] have shown that droplet groups are less affected by ambient airflow shortly after exhalation due to turbulence, and even large droplets can travel farther. The concentration of bioaerosol particles reached a stable state after being released into space for 400 s [53]. Under ventilated conditions, small droplets and droplet nuclei in the air can spread to a long distance with the airflow. More complex deposition and resuspension phenomena also occur during this process. In addition, environmental factors such as AT, RH, ultraviolet radiation (UR), and surface materials can also affect the virus viability during transmission.

### 3.3. Exposure and Infection

There are generally four ways of virus exposure as follows: (1) respiratory fluid exchange between facial membrane, (2) RDs deposition by direct splash on exposed facial membrane, (3) touching facial membrane with hands contaminated with RDs or virus-bearing surfaces, and (4) inhalation of fine RDs and droplet nuclei. 

The virus can cause infections in different organs of the body, but the relationship between the location of virus deposition and the mechanism of infection remains unclear. In the first three exposures, the viral particles can trigger an infection once they meet the facial membrane. For the fourth, inhaled particles can deposit on the surface of the respiratory tract and cause infection. The nose is reportedly more sensitive to SARS-CoV-2 than the mouth and eyes [54]. Larger particles >10 μm generally settle in the nasopharynx and particles smaller than 5μm can be directly inhaled into the alveoli [55]. The infective dose, i.e., the number of particles that lead to an infection, is highly dependent on virus biological data, which is generally difficult to obtain early in an epidemic. Karimzadeh et al. [56] estimated that the minimum infective dose of COVID-19 in humans is higher than 100 particles. Additional studies are needed to complement epidemiological data that provide insights into the dose-response relationship between received doses and disease outcomes for different exposures.

### 3.4. Classification of SARS-CoV-2 Transmission Routes

COVID-19 is thought to spread mainly through close contact, which is defined as any full or partial face-to-face interaction with an infected person within 1.5 m [57]. During close contact, small droplets and droplet nuclei are considered to dominate rather than large droplet deposition [58]. The total projected surface area of the membranes of the eyes, nostrils and lips is ~15 cm^2^, only about 1.2% of the frontal area of the head [59], where only very few droplets are deposited directly. The intensity of exposure of the susceptible individual to droplets 50–100 µm and smaller than 50 µm within 1 m is 487 and 13,898 times that due to direct deposition when the infected person talks, respectively, [15]. The significant indoor air and surface contamination by patients with SARS-CoV-2 through respiratory droplets suggests that aerosols and fomites are potential mediums of virus spread [5]. Guidelines and public health information have acknowledged that aerosol transmission occurs under certain circumstances, especially over long periods in crowded, inadequately ventilated indoor spaces [60]. Updated WHO guidelines state that people can become infected by touching surfaces contaminated with the virus and by touching their eyes, nose or mouth without cleaning their hands [1]. The CDC also stresses the importance of frequently disinfecting surfaces and objects that are touched by many people [57]. Multiple studies have found viral RNA in stool samples of patients with COVID-19 [61]. The outbreak of SARS in Amoy Gardens in Hong Kong [62] and the community outbreak of COVID-19 in a high-rise building in Guangzhou [63], was presumed to be related to faecal-oral transmission and faecal-aerosol transmission, respectively. Yet evidence in the current study to support rectally shed SARS-CoV-2 is infectious remains weak, only few reports in the literature support retained infectivity of rectally shed SARS-CoV-2 [64]. Faecal-related transmission is considered unlikely to be a primary route for propagating the infection [64]. Therefore, faecal-oral transmission is not included in the following analysis.

To carry out the subsequent analysis more clearly, the SARS-CoV-2 transmission routes are classified into 6 types in this paper according to interpersonal distance and exposure way (Table 2), including 4 types of short-range exposure:

1. Immediate physical contact, including face, hands, and other exposed skin;
2. Personal fomite contact, including clothes, glasses, hats, ties, telephones, and other items passed by hand;
3. Face-to-face contact, including being coughed or sneezed on or talking within 1 m.
4. Having been within 1.5 m without face-to-face contact,

and; 2 types of long-range exposure:

1. Contact contaminated surfaces, such as door handles, elevator buttons, and water dispenser buttons;
2. Inhalation of contaminated aerosols.

Note that multiple routes of transmission may occur simultaneously. For example, in addition to direct contact, a handshake may involve a short-range airborne route, or even a large droplet route, if accompanied by a conversation. 

## 4. The Role of Spatial Factors on SARS-CoV-2 Transmission

### 4.1. Distribution of SARS-CoV-2 in Indoor Spaces

Positive samples for SARS-CoV-2 RNA can be detected in rooms with infected individuals, including in the air and on the surfaces of buildings and their personal belongings [65]. Aerosols containing different concentrations of the virus have been detected in the toilets, medical-staff areas, ventilated wards, and isolation rooms used by patients in hospitals [66,67]. The longer a COVID-19 patient stays in a space, the more likely they are to touch and, thereby, contaminate environmental surfaces. As shown in Table 3, compared with non-hospital settings, a higher number of contaminated surface samples were detected in hospitals, but a lower percentage of samples tested positive [68]. The most contaminated spaces in hospitals were the COVID-19 intensive care unit (ICU), COVID-19 maternity isolation ward and general isolation ward, followed by outpatient lobbies and emergency departments [69]. The non-hospital setting has a smaller overall sample size, with stores having the highest proportion of positive samples, followed by banks and quarantine hotels [70,71], where more testing is needed. We classify indoor surfaces susceptible to contamination into three categories: building envelopes, public facilities, and personal belongings, and counted their positive samples separately. The public facility surfaces had the highest rates of contamination, especially doorknobs, and viral RNA-positive samples were detected on public facilities in almost all spaces. Other contaminated facilities were related to the type of space, such as toilet seats in toilets and basket handles in shops. In addition, the most contaminated personal items were drinking glasses, cell phones and gloves. 

The virus distribution that was detected is due to a combination of droplet deposition and occupant touch. Liu et al. [53] explored the diffusion and deposition of bioaerosols using laboratory experiments and numerical simulations and found that almost 70% of the particles were deposited on building surfaces, including walls, ground, ceiling, furniture, and equipment. However, few positive samples from walls and floors were detected (Table 3), which may be due to the decline and resuspension of the virus or the location of sampling. Research shows that personal items were most frequently touched, followed by public facilities (including furniture and equipment), while floors, walls and ceilings were rarely touched [72]. Personal belongings (such as mobile phones) may be more likely to carry the virus but less likely to transmit the virus due to infrequently touched by others. Since the distribution network of the virus is not consistent with the touch network of the occupants, the spread based on environmental fomites is not only related to the touch behavior of the occupants, but also to the distribution and deposition of aerosols.

Spatial features can reflect individual attributes (role, gender, age) and behavioral characteristics (type of activity, activity level, motion state, and duration), thus potentially influencing the indoor infection process. Taking the school as an example, its functional space mainly includes classrooms, offices, dormitories, stairwells, corridors and lavatories. Table 4 shows the six typical space-related occupants and their activity characteristics, as well as possible contagion-related behaviors and transmission routes. Classrooms and offices are mainly used by teachers and students, who are densely distributed in fixed positions for a long time (>40 min) in order to keep sitting, and who may interact with other people and environmental surfaces. Almost all the transmission routes discussed above may occur in these spaces. Although there are only a few students in a dormitory, they are prone to close contact due to their close relationships and spend more than seven hours in a space, which puts them at high risk of infection. On the other hand, stairwells and corridors are traffic spaces that connect classrooms, offices, etc., where most people walk through and for short durations, yet possible congestion during peak hours greatly increases the chances of getting in touch with various people. Door handles, flush buttons and faucets in the lavatory are frequently touched by different people, and the risk from surface contact requires more attention. In addition, people walk at different speeds according to spatial characteristics and travel purposes [73], resulting in different exposure times in the same length of space. Walking speeds were fastest in shopping centres, followed by offices and leisure halls, and slowest in stairwells.

### 4.2. Spatial Factors Involved in SARS-CoV-2 Transmission

**Figure 3:**
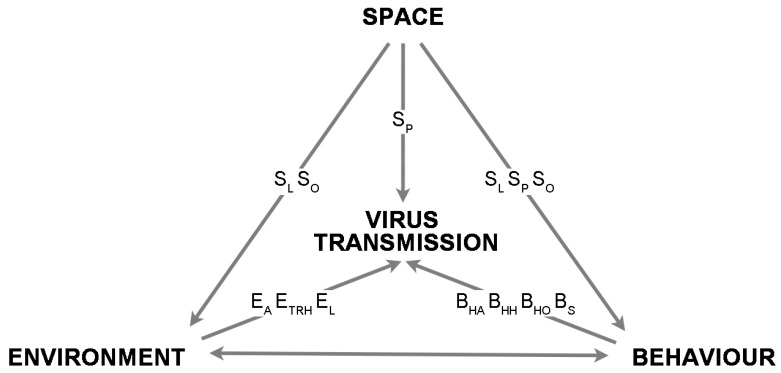
Relationship between space, environment and occupant behavior parameters in the virus transmission process. (An uncomfortable indoor environment may drive adaptive or controlling behaviors, such as adding or removing clothing or turning the air conditioner on and off. Although these behaviors are usually accompanied by some surface contact, the risk of these exposures is extremely low and therefore not included in the scope of this study).

Although many studies deal with the impact of spatial factors on indoor environmental quality (IEQ) and occupant behavior, only a few studies are related to the relationship between viral spread and spatial parameters. Table 5 lists the spatial, environmental, and behavioral parameters that affect virus transmission. The space design parameters can be divided into three categories: plane layout design, such as space configuration and physical layout; public facility design, such as touchless technology and surface material selection; and opening design, such as doors and windows. The triangular relationship between space, environment, occupant behavior, and virus transmission is summarized in Figure 3. The impact of spatial factors on virus transmission includes two aspects: direct influence on virus viability through surface material (S_P_-V_V_), and indirect influence on virus spread and viability through intervention in human behavior and IEQ. The plane layout not only determines the path and relative position of occupants, but also affects the airflow field of the room, thereby interfering with the probability and degree of human-to-human and human-to-object interaction (S_L_-B_HH_/B_HO_-V_S_), as well as the distribution of virus concentration in the air (S_L_-E_A_-V_S_), respectively. The touch-free design of public facilities helps reduce user contact with contaminated surfaces (S_P_-B_HO_-V_S_). Door and window openings determine indoor ventilation patterns (mainly natural ventilation) and thus affect the spread of virus-laden air (S_O_-E_A_-V_S_). In addition, the window configuration can change the temperature, humidity, and daylighting conditions in local areas of the room, thus affecting the stability of the virus (S_O_-E_TRH_/E_L_-V_V_).

### 4.3. Spatial Effects on Virus Droplet-Based and Surface-Based Transmission

Human-to-human transmission depends largely on people’s behaviors, especially when in close contact. The relative location to the infected person and gesture determines the manner of exposure. The details of respiratory activities and touching contaminated surfaces influence exposure levels, including exhalation and inhalation of virus particles and their transfer between contaminated surfaces, hands, and faces (mouth, nose, and eyes). A comprehensive understanding of human transmission-related behavior helps to assess the infection risk comprehensively and take corresponding preventive measures.

#### 4.3.1. (S_L_-B_HH_/B_HO_-V_S_) Spatial Layout Affects Human-To-Human and Human-To-Building Interactions

**Figure 4:**
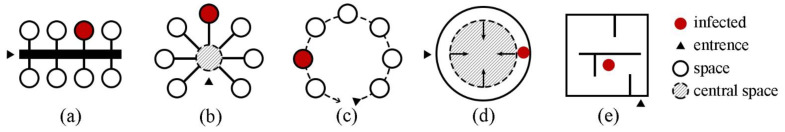
Basic spatial organizations. (**a**) corridor-based combination; (**b**) hall-based combination; (**c**) tandem combination; (**d**) wraparound combination; (**e**) free combination.

The impact of spatial layout on occupant interactions is critical to the spread of COVID-19 in buildings. For example, spatial arrangements based on open-office concepts can enhance collaboration and communication among workers but increase the opportunities for virus transmission. A proper spatial organization has a redistribution effect on pedestrian flow, allowing people of different identities to move independently of each other, reducing route interference, and avoiding congestion-prone nodes. Figure 4 shows five basic spatial organizations. The corridor-based combination, which separates the main space and traffic space so that the rooms do not interfere with each other, is commonly used in dormitories, office buildings, schools, and hospitals. The hall-based combination takes the hall as the crowd flow centre, and other spaces are directly connected with the hall without interfering with each other. It is suitable for public buildings such as libraries and railway stations. The tandem combination is to connect the space directly without using special transportation space, which has strong continuity and clear use procedures and is suitable for museums. The wraparound combination refers to the form of a large space as the centre, surrounded by small ancillary spaces, which is suitable for buildings with prominent main spaces, such as theatres, cinemas, and gymnasiums. The free combination means that a unified large space is separated into several parts flexibly, which have strong connectivity and no clear boundaries. Most exhibition halls adopt this combination. Large public buildings usually adopt more than one spatial combination form to accommodate complex functions and a large numbers of people. The different spatial configurations show differences in virus diffusion, and its effect on the diffusion of polluting gases is more pronounced than that on the diffusion of viruses on surfaces. Compared with linear structures and centralized structures, fractal structures exhibit higher diffusion efficiency [74].

**Figure 5:**
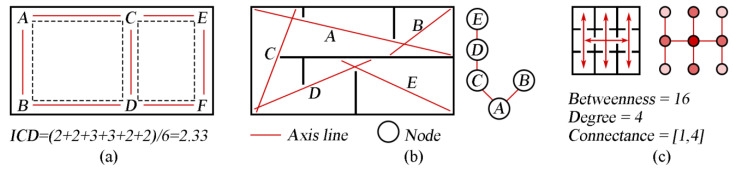
(**a**) The mean ICD value (A = 2, B = 2, C = 3, D = 3, E = 2, F = 2, adapted from Reference [78]); (**b**) a schematic diagram of axial lines and justified graph (adapted from Reference [78]); (**c**) spatial layout and microbial abundance. The arrows represent possible directions of microbial spread and darker colours represent higher microbial abundance. Betweenness (the number of paths that go through the space), degree (the number of doors in the space), connectance (the number of doors between any two spaces) (adapted from Ref. [79]).

The effectiveness of the user’s experience of positioning and routing in a building is related to the familiarity of the space (relative to the role) and the complexity of the spatial organization. Most people tend to take the shortest route with the fewest changes in direction to their destination. A simpler spatial organization, that is, the shorter overall path length, exhibits a higher degree of human interaction [75]. O’Neill [76] proposed the concept of interconnectedness density (ICD), which represents the number of passable paths between choice points. Figure 5a, shows the calculation of the mean ICD value. The larger the value, the more complex the spatial sequence. Since the characteristics of space itself are less considered, spaces with different planes may share one ICD value. The space syntax proposed by Bill Hillier [77] can be used to predict the possible impact of space on users by decomposing space into components and analysing their accessibility, connectivity and integrality. As shown in Figure 5b, axial lines are the longest and fewest lines covering all convex spaces, which are treated as nodes, and their interrelation can be abstracted into a justified graph [78]. Based on this, Coil et al. [79] used betweenness, degree and connectance indexes to analyse spatial configuration (Figure 5c). Integrated and intelligible structures have been shown to enhance movement volume in more accessible and central places, as well as the number and variety of microbes [79]. Abdul et al. [80] quantified the impact of spatial permeability and wayfinding levels (Table 6) on COVID-19 transmission and social distancing using an analysis method of space syntax, and the results showed that clear and direct spatial organization was associated with more effective social distancing. Compared with the grid plan, the linear-plan layout is considered to reduce the risk of user crossing due to its convenience of pathfinding. Efficient designs that reduce entrances and user access can help to meet COVID-19 safety requirements.

**Figure 6:**
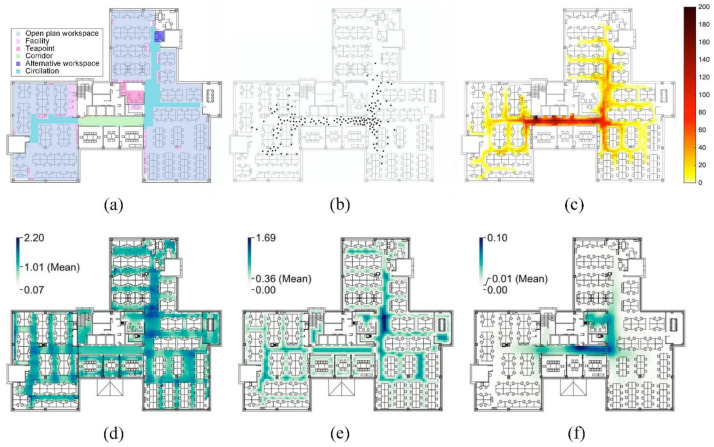
(**a**) Floor plan with functions; (**b**) Scenario snapshot (t = 30 s); (**c**) Heat map; (**d**) Visual control (the space visible from a cell to the other directly visible cells); (**e**) Isovist min radial (the distance from the Isovist origin to the nearest obstacle); (**f**) Travel concentration (the effect of attractors) [81].

Additionally, the micro-pedestrian simulation in buildings can reproduce the walking behavior characteristics of people in real scenes based on pedestrian dynamics, thereby testing the effectiveness of different physical layouts. Pan et al. [81] investigated the impact of spatial design on social distancing by simulating movement in an open office and compared some results with spatial configuration (Figure 6). Corridors are found to be high-density moving areas with relatively high indoor travel concentration and Isovist min radial value, which are mainly determined by the location of elevators, stairs, entrances and exits. Meeting rooms, restrooms and public facilities around the circulation area are also relatively intensive points of movement. The tea point is rarely visited possibly due to the low level of visual control. For moving individuals, public facilities are regarded as attractors or obstacles, thus interfering with their path of movement and frequency of use. They usually change direction and speed 1.5–8 m in advance to actively avoid obstacles [82]. In addition, studies have shown that increasing the availability of hand cleaning and sanitizing equipment can increase the probability that they will be used [83]. Arranging these rooms and public facilities properly can help divert traffic and reduce centralized visits.

**Figure 7:**
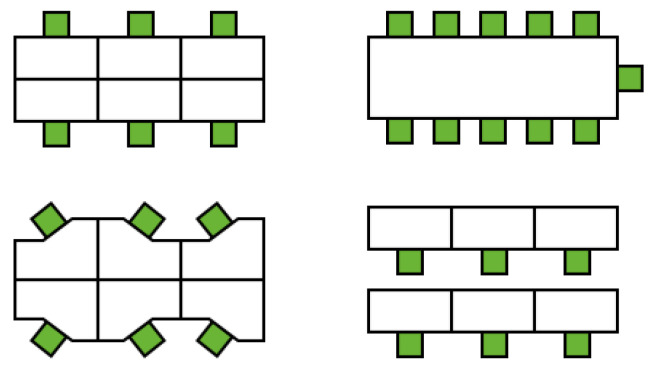
The arrangement of tables and chairs.

**Figure 8:**
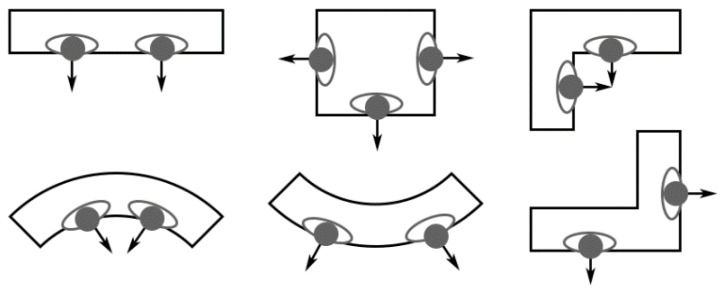
The form of the seat and the position of the occupant.

**Figure 9:**
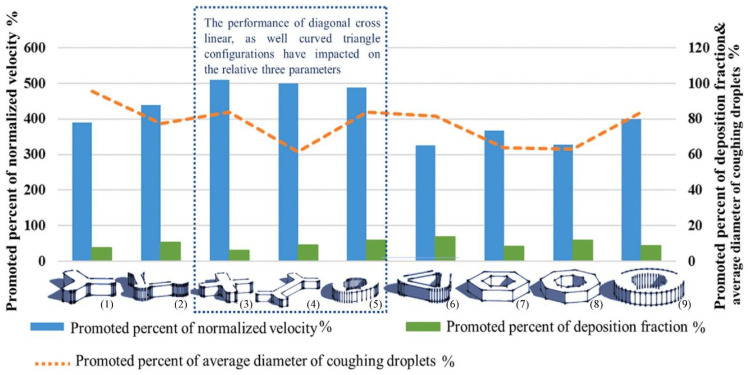
The effect of seat configuration on the normalized air velocity, deposition fraction, and average droplet diameter [84].

The arrangement of furniture, especially tables and chairs, determines the relative position of people when they sit for long periods (classes, offices, meetings, etc.), and is crucial to maintaining social distancing. For spaces with fixed seating (such as dining room, office, classroom, etc.), the number and spacing of tables and chairs represents the density, relative distance and the angle of the crowd (Figure 7). As shown in Table 4, when fully loaded, the crowd density of the classroom is larger than that of the office. For the leisure space, the seating forms are designed in a variety of ways, which can affect the occupant’s position and orientation (Figure 8). Square and convex seats help reduce face-to-face conversations compared to long and recessed seats. Hassan et al. [84] evaluated the effect of nine seat designs on droplet diameter and deposition (Figure 9) and showed that seats (1), (3), and (5) reduced droplet movement, and diagonal-cross linear and curvilinear-triangle configurations helped reduce droplet deposition.

#### 4.3.2. (S_P_-B_HO_-V_S_) Touchless Design for Public Facilities Helps Reduce Exposure to Fomites

The COVID-19 pandemic has brought unprecedented attention to touchless technology and has accelerated its development and use. In daily life and work, people inevitably touch certain private or public surfaces such as banknotes, doorknobs, and elevator buttons. Disinfection is required immediately after each contact, but this is difficult for most people to fully do. Touchless interaction technology is a new solution that can help reduce infection caused by exposure to fomites. 

Table 7 shows the common touchless technologies and their applications for adjacent people-object interaction. Infrared sensors can use infrared rays for data processing and control the operation of driving devices and are widely used in automatic doors, lights, toilets, faucets, disinfectant dispensers, and hand dryers, which allow public places to be more hygienic. [85]. Biometric systems can identify individuals based on their biological characteristics to allow specific groups of people to interact with computers, which includes facial recognition, iris recognition, voice recognition, and hand recognition [86]. Traditional fingerprint recognition systems with hygiene risks now have a new solution, namely contactless fingerprint recognition technology [85]. Wireless communication technologies (such as RFID [87], NFC [88] and QR code [89]) allow short-range touchless data exchange through personal devices, such as opening doors with access cards, Apple Pay payments and scanning QR codes with mobile phones. At present, the most widely used infrared sensing technology cannot cope with the needs of high precision and complex instructions. Therefore, real-time gesture interaction and eye tracking may be the future exploration direction of touchless interaction technology.

### 4.4. Spatial Effects on Virus Air-Based Transmission

#### 4.4.1. (S_O_-E_A_-V_S_) Opening Design Determines Airflow Field Due to Natural Ventilation

Spatial openings generally refer to windows and doors, both of which are related to indoor-airflow organization caused by natural ventilation. The position of the window openings in the horizontal and vertical directions is the main factor affecting the airflow pattern, which is usually studied through field measurements and numerical simulations. Zhou et al. [91] investigated the influence of the position of the window opening on the effective ventilation of the room and found that ventilation rates reached the minimum when the window opening was located at the centre of the windward face. Vertically, the further the window is from the centre of the windward side, the greater the ventilation [91].

**Figure 10:**
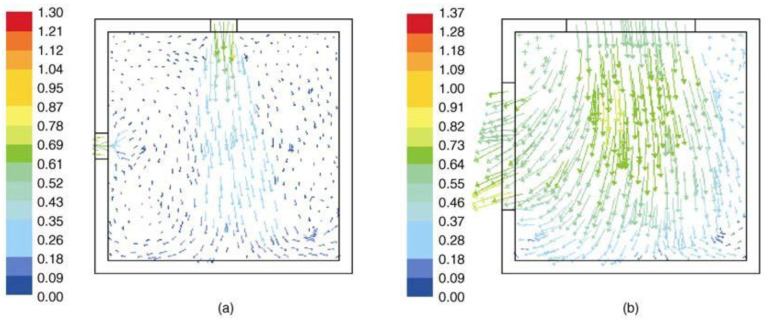
Velocity vector (m/s) plot at 2.5 m height. (**a**) WWR = 0.01; (**b**) WWR = 0.25 [92].

**Figure 11:**
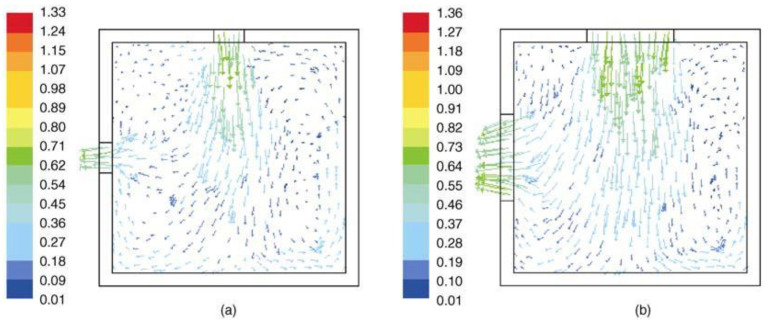
Velocity vector (m/s) plot at 2.5 m height. (**a**) AR = 0.36; (**b**) AR = 2.78 [92].

The size and shape of the window opening also have a significant influence. As shown in Table 8, Ravikumar et al. [92] compared the mass flow rate (MFR) and wind speed from CFD models with different window-wall ratios (WWR) and aspect ratios (AR). The MFR and wind speed increase with the increase of WWR (Figure 10). The change of AR affected the local airflow field and had no obvious influence on the overall MFR (Figure 11). Additional numerical simulation results show that the ventilation efficiency is the best when the AR value is 4 [93].

**Figure 12:**
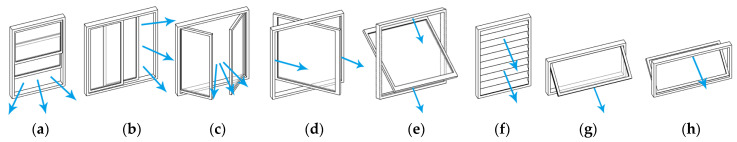
Typical windows and natural ventilation. (**a**) VSW (**b**) HSW (**c**) TURN (**d**) VPW (**e**) HPW (**f**) BLIND (**g**) AW (**h**) TILT. (The blue arrows indicate the direction of natural ventilation).

**Figure 13:**
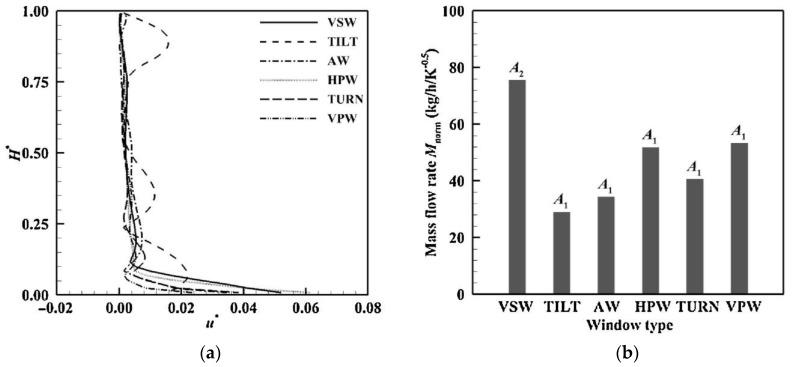
(**a**) Dimensionless velocity profiles; (**b**) normalized mass flow rates [94]. (Physical open area *A*_1_ = 0.2178 m^2^, *A*_2_ = 0.2214 m^2^).

In addition, in the case of similar physical openings, the characteristics of incident wind and indoor airflow vary with the configuration of windows, which have been generally simplified to rectangular openings and have rarely been studied quantitatively [94]. Figure 12 shows seven typical windows: (a) vertical-slide window, (b) horizontal sliding window, (c) turn window, (d) vertical-pivot window, (e) horizontal-pivot window, (f) blind, (g) awning window, and (h) tilt window. Wang et al. [94] investigated the ventilation of six kinds of windows (excluding (b) and (f)) through numerical simulation. As shown in Figure 13, the air velocity in the vertical direction did not change significantly for most of the cases, except for the Tilt window. VSW provides the maximum MFR, followed by VPW and HPW, while TILT provides the minimum ventilation rate, which is less than half of the VSW. 

**Figure 14:**
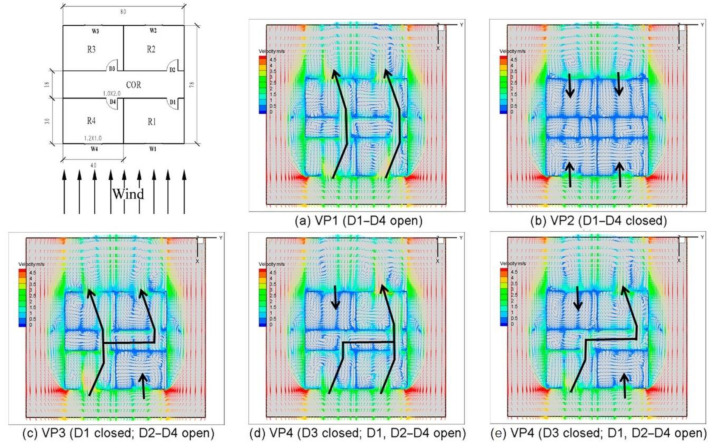
The velocity distribution and the airflow patterns under five ventilation paths (z = 1.2 m, WWR = 0.1) [95].

In a multi-room building, the opening of the doors facilitates air convection and thus increases the ventilation rate of the room, which is thought to be effective in diluting contaminated air. As shown in Figure 14, five ventilation paths are formed by controlling the opening and closing of the four doors (D1, D2, D3, D4) [95]. A complete cross-ventilation path is established under VP1, providing ample fresh air to every room from inlet to outlet. In VP2, the four rooms are all ventilated on one side, and the indoor-airflow is very weak, which is the most likely to cause uneven air mixing. Under VP3, air flows from R4 to R3 and R2 with inflow rates of 62.2% and 37.8%, respectively. The higher velocity of the VP4 air outlet than VP5 is due to the opening of R1, which, in turn, leads to the difference in the airflow distribution of R3.

#### 4.4.2. (S_L_-E_A_-V_S_) Physical Layout Affects the Distribution of Virus Concentration

**Figure 15:**
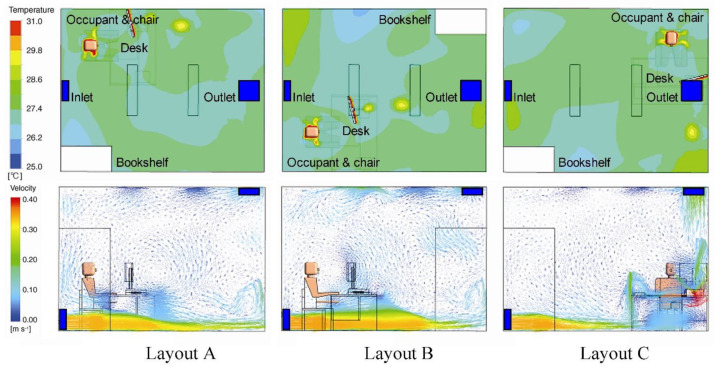
Temperature fields (z = 1.15 m) and airflow fields (y = 1.6 m) of three typical layouts of office room (DV2) [96].

Changing the physical layout at room level has been shown to cause changes in indoor-airflow fields, which, in turn, induce changes in ventilation effectiveness and distribution of pollutants in the air. Zhuang et al. [96] simulated the airflow fields of three furniture layouts of the office room (Figure 15) and calculated their ventilation effectiveness under four ventilation modes according to changes in volatile organic compound concentrations (Table 9). The results show that changing the position of the furniture can significantly improve the air quality in the breathing area under either mixed ventilation or displacement ventilation. Layout B has the best overall ventilation by keeping occupants away from pollution sources and closer to the air inlets [96].

### 4.5. Spatial Effects on Virus Viability

#### 4.5.1. (S_P_-V_V_) Virus Viability Varies with Surface Material of Indoor Public Facilities

Although SARS-CoV-2 RNA has been widely detected in the real environment, little has been said about the materials on exposed surfaces of spaces and facilities, which affect the stability of the virus on them. The viability of SARS-CoV-2 on different materials was mostly evaluated under laboratory conditions as shown in Table 10. At room temperature (RT), RH 65%, SARS-CoV-2 is stable on stainless steel and plastic, surviving for 3–7 days, followed by glass, wood and clothing (1–4 days) [97], and quickly inactivated on copper and paper (less than 4 h and 24 h, respectively) [82]. Studies by Harbourt et al. [98] have shown that the virus can survive for more than 8 h on skin samples (exposed at 37 °C). The virus titer decayed exponentially under all experimental conditions. Survival rates of SARS-CoV-2 measured by Riddell et al. [99] using standard ASTM E2197 substrates simulating human secretions were significantly prolonged on all surfaces. Surface roughness also plays a role in the virus transfer between the hand and the surface. Fabric/porous material has a higher transfer rate than nonfabric/nonporous material in general [100]. Note that experiments use very high concentrations of virus out of necessity, which is not encountered in natural conditions.

A few studies have examined the effect of antimicrobial coatings on SARS-CoV-2. Behzadinasab et al. [101] found that the virus half-life on cuprous oxide coating was only 3–4 min, and 99.9% could be inactivated within 1 h without being affected by humidity. Hutasoit et al. [102] found that the reduction rate of the virus on cold sprayed-copper coating was more than 90%. Hosseini et al. [103] found that porous CuO coating with 50 μm could reduce infectivity by 99.7% in 1 min. Most of these coatings are based on copper materials, and their fabrication and application are of great significance in reducing the transmission risk of high-frequency touch surfaces. 

#### 4.5.2. (S_O_-E_TRH_-V_V_) Window Openings and Physical Layout Change Indoor AT and RH

Spatial factors related to the inhomogeneity of indoor AT and RH include window openings and physical layout, which influence virus viability on surfaces and in the air [104,105]. The long-wave radiation of the façade exposed to the sun causes local spatial thermal environment changes and when the window is closed, the variation in solar radiation through the glass results in a temperature difference of more than 4 °C with WWR from 0.6 to 0.9 [106]. In addition, Figure 15 shows that the physical layout, especially the location of the heat source, also has a certain influence on the temperature field. However, the shading device can effectively reduce the solar heat absorbed by the window, so that the indoor thermal conditions are more uniform. Furthermore, opening windows greatly contributes to enhancing the heat exchange between indoor and outdoor and reducing their temperature difference [106]. 

Indoor AT and RH changes not only affect the evaporation and trajectory of virus-containing droplets (Section 3.1), but also the stability of the virus on surfaces and in the air. It took 14 days for SARS-CoV-2 to completely lose infectivity in the viral transport medium at RT, and it can be stable at 4 °C for more than 14 days, but undetectable in less than 5 min at 70 °C [97]. As shown in Table 11, more studies focus on the viability of SARS-CoV-2 on surfaces at different AT and RH. Studies have shown that increasing temperature significantly accelerates virus inactivation on the surface, while there is no conclusion on the optimal conditions for the survival of SARS-CoV-2 [71,99,107,108,109]. When the RH was 50%, the SARS-CoV-2 survived on stainless steel for 28 days, 7 days and 2 days at 20 °C, 30 °C and 40 °C, respectively, [99] and the survival time of the virus on plastic was reduced from 4 days to about 10 min when the temperature increased from 22 to 70 °C [71]. However, Kratzel et al. [110] reported no significant difference in the stability of SARS-CoV-2 on metal discs from 4 °C to 30 °C, and this was hypothesized to be due to the lack of strict control over other environmental variables such as humidity and light. However, there is controversy regarding the effect of humidity on virus viability. Biryukov et al. [108] found that an increase in RH from 20% to 80% accelerated virus inactivation. While Morris et al. [107] observed that the virus was more stable at RH40% and 80% than at 65%. Similar properties have been reported for other coronaviruses, which survived for the longest time at RH 50% than lower or higher RH (20%, 30%, and 80%) at 20 °C [111]. In addition, Matson et al. [112] found that the half-life of the virus in artificial saliva aerosols and tissue culture aerosols showed opposite trends with increasing humidity at RT. 

The above data are derived from limited laboratory experiments in which mechanistic models of viral decay kinetics were developed to predict the unobserved effects of AT and RH [107,108]. Many studies have been performed on the role of architectural design elements in the indoor thermal environment, and further studies on the survival of SARS-CoV-2 in real environments are needed to help quantify the impact of spatial parameters on it.

#### 4.5.3. (S_O_-E_L_-V_V_) Daylighting Introduced by Windows Accelerates Virus Decay

Window orientation, WWR, window type (visual and solar transmittance) and shading devices determine daylighting quality, area, and duration [113], and solar radiation plays an important role in the spread of COVID-19 [114]. An et al. [115] studied the effect of simulated sunlight on the stability of viruses in aerosols, suggesting that the rate of virus decay can be accelerated. In addition, studies have shown that sunlight can also rapidly inactivate viruses on inanimate surfaces [116]. UV consists of UVA (315–400), UVB (280–315), and UVC (100–280). UVC has been found to effectively inactivate viruses including SARS-CoV-2 [117], although UVC in sunlight has difficulty penetrating the ozone layer and reaching the ground. UVB also appears to be able to inactivate viruses present on surfaces and in the air to some extent [118]. Furthermore, studies have shown that in the absence of photosensitizers, the increased susceptibility of the virus to visible light at 405 nm mediates inactivation [119]. Based on the available data, it can be assumed that natural light can disinfect air and surfaces. Thus, increasing the WWR of south-facing windows, or properly reducing unnecessary shading to increase the level and duration of sunlight exposure may help accelerate virus decay and prevent the spread of disease. Further research is needed on the effect of window parameters on virus inactivation through the introduction of sunlight. 

## 5. A Space-Based Framework to Assess the Indoor Infection Risk

Numerous mathematical models have been developed to calculate the human infection risk [120,121]. Wells–Riley and dose-response approaches are widely used to assess infection risk [122]. The Wells–Riley model is based on the concept of quanta (a quantum is defined as the number of virus particles required to infect a person) and can only be used to assess airborne transmission risk [123]. The improved Wells–Riley model can be combined with computational fluid dynamics (CFD) simulations to improve the spatial resolution of infection risk distributions. Yan et al. [124] developed a 3D predictive model to analyse airborne transmission risks in airliner cabins based on the Wells–Riley framework coupled Lagrange particle simulations. Srivastava et al. [125] developed a Eulerian CFD model to assess the infection risk of COVID-19 in office buildings in combination with the Wells–Riley model. The dose-response model requires dose-response relationships, which are derived from large amounts of infectious dose data [126]. These data are difficult to obtain in the early stages of an outbreak, but can also be inferred from data from other related viruses and animal experiments. The dose-response model is divided into a deterministic model and a stochastic model, the latter of which allows consideration of stochastic characteristics of virus exposure and intake [122]. Buonanno et al. [127] proposed a quantitative approach based on the dose-response model to estimate the risk of airborne transmission of SARS-CoV-2 infection by dividing the whole process into evaluation of the emission rate, exposure to a dosage concentration, received dosage and infection probability calculations.

Spatial factors have a great potential impact on the indoor virus transmission process, but these impacts have not been fully reflected in the assessment of indoor infection risk so far. There are several limitations to current studies on indoor COVID-19 risk assessment in considering the spatial factors: (1) First, the influence of building space factors on indoor infection risk is rarely discussed, and is mostly regarded as a “background” to analyse the effects of other factors such as behavior management, ventilation rate, etc.; (2) Second, most indoor infection risk assessment models do not integrate multiple transmission routes in the building space, but only focus on a single transmission route; (3) Third, the dynamic interaction between people and the environment were often oversimplified because the influence of spatial elements on occupant behavior is not taken into account. 

**Figure 16:**
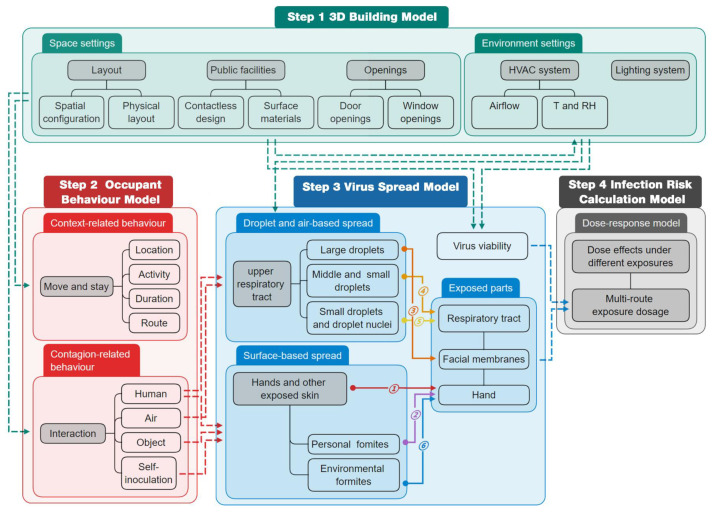
Multi-route infection risk assessment framework considering spatial, environmental, and behavioral factors. (*①* Direct contact route, *②* Personal fomite route, *③* Large droplet route, *④* Short-range airborne route, *⑤* Environmental fomite route, *⑥* Long-range airborne route).

Therefore, we proposed a space-sensitive, multi-route infection-risk assessment framework (Figure 16), which includes four parts: a 3D building model, an occupant-behavior model, a virus-spread model and a risk-calculation model. The framework can be used to assess the impact of spatial, environmental, and behavioral factors on SARS-CoV-2 transmission and the effectiveness of different prevention and control measures.

### 5.1. 3D Building Model

Given the significant impact of spatial parameters on occupant behavior and virus transmission, an integrated 3D building model is the basis for simulating building-level COVID-19 outbreaks. The model contains detailed architectural space information, such as walls, doors, windows, tables and chairs, etc., which have specific material and touchable properties. In addition, the model includes indoor environmental information (thermal and light conditions) from the integration of building spatial parameters and HVAC parameters.

### 5.2. Occupant Behavior Model

**Figure 17:**
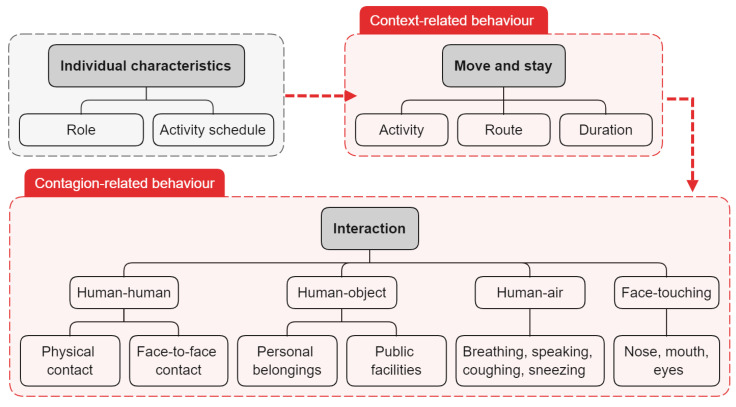
Detailed composition of occupant behavior model.

Since occupant behavior is influenced by the interaction of the body’s internal needs with the external environment, the occupant behavior model is based on individual characteristics and a detailed indoor-space model. As shown in Figure 17, in the process of indoor respiratory-disease spread, the occupant-behavior model includes context-related behaviors (activity type, moving path, and duration) and contagion-related behaviors (human-human interaction, human-object interaction, and human-air interaction).

#### 5.2.1. Context-Related Behavior

Context-related behaviors include location, activity, movement route and duration, which are highly correlated with architectural spatial characteristics. The roles of occupants and their schedule of activities are determined according to the building type. Then, a dynamic grid of the spatiotemporal sequence of individual activities is established, which mainly includes two states: move and stay. A given stay state contains several activities and their durations, and a given move state contains the speed and path of movement based on purpose and context. In addition, multiple individuals in a space at the same time may exhibit some group-behavior characteristics (such as crowding and queuing) due to the intersection of paths. Taking a store as an example, the basic activity process of a customer includes entering the store, picking items, checking out, and leaving the store, and its arrival time, shopping duration and path are of great uncertainty. The basic activity process of a cashier includes arriving at the store, providing cashier services, and leaving the store, with a clear commute time and work schedule. There will be peaks in traffic at certain times, resulting in possible queues for customers to enter the store and check out.

Extensive literature has discussed human activities observed in public spaces. Taking the office buildings as an example, sitting time usually accounts for more than 60%, standing time accounts for 10–20%, and stepping time is about 7–12% [128]. The average frequency of workers leaving their seats is 1.6 times per hour, with 0.38 and 0.96 trips to the restroom and kitchen, respectively, [129]. Students were observed to spend an average of 1.36 h in the office [130]. The collection and analysis of behavioral data in different functional spaces (positioning systems, video recordings, questionnaires, etc.) allow for high fidelity and randomness of dynamic context-related behavioral simulations.

#### 5.2.2. Contagion-Related Behaviors

**Figure 18:**
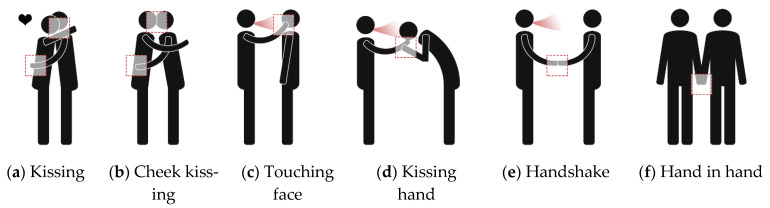

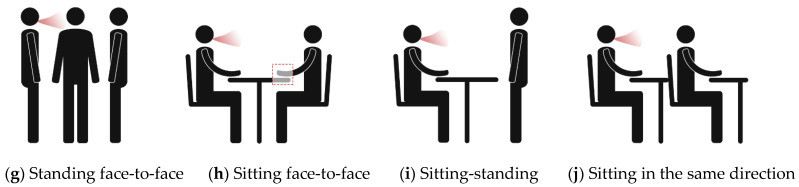
Schematic diagram of human-human interactions.

Contagion-related behaviors are interactive behaviors performed by occupants in the process of moving and staying that promote the spread of the virus. It contains human-human interaction, human-object interaction, human-air interaction and self-inoculation. As shown in Figure 18, different interactions lead to different transmission routes, which in turn lead to different ways of exposure. 

**Figure 19:**
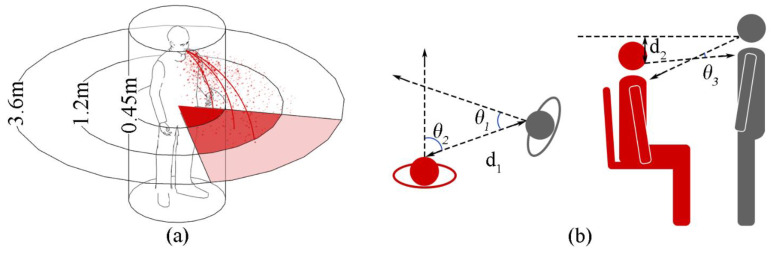
(**a**) Personal spaces and their risk levels; (**b**) Close contact parameters. (d_1_: horizontal distance, d_2_: vertical distance; θ_1_, θ_2_: the horizontal angles between the breathing direction of the infected and the susceptible; θ_3_: The vertical angle between the breathing direction of the infected and the susceptible).

Human-human interaction includes direct physical contact (Figure 18a–f) and close contact (Figure 18c–j). Edmunds et al. [131] divided human contact into four types: sexual contact, including kissing accounted for only 2% (not included in the scope of this paper), conversation with non-sexual physical contact accounted for 34%, physical contact without conversation accounted for 3%, and conversation without physical contact accounted for the largest proportion, up to 61%. Except for kissing, which is transmitted through body fluids, all other types of direct physical contact are transmitted from skin to hands and then to the facial membranes. Hall et al. [132] proposed the concept of Proxemics and divided personal space into four categories: private zone, personal zone, social zone and public zone. Table 12 shows the social relationships between people, the possible interaction behaviors and distance corresponding to the four types of personal space. Furthermore, in a room with a specific physical layout, the type of activity also has an impact on the possible relative positions of two persons [133]. Table 13 shows the effect of the activity type on seat selection. It has been observed that people tend to sit face-to-face or next to each other when talking, face-to-face or on opposite sides of a table when collaborating, and face-to-face or diagonally when competing [134]. As shown in Figure 19a, the risk of interaction increases as horizontal distance decreases within a certain range in front of an infected individual (the area of short-range droplet spread),. Parameters related to direct physical contact include contact location, area, force, and duration. Figure 19b shows the parameters related to human interaction, including the horizontal and vertical distances and angles between the faces of the infected and susceptible [15]. Most studies assumed full face-to-face interaction, where two people are of the same height with θ_1_ and θ_2_ values of 0.

Human-air interaction refers to respiratory activity. Exhalation activities (Table 1) include breathing, speaking, coughing, sneezing, etc., which mainly affect the emission rate and the farthest horizontal distance of virus-containing droplets. Coughing is one of the most common symptoms of COVID-19. When a healthy individual was infected, he tends to cough continuously, and the number of particles emitted by one cough increases [135], and the particle diameter becomes slightly larger [32]. Studies have shown that normal breathing produces more viral aerosols over time than coughing due to the high frequency, though more infectious droplets are ejected through a cough and spread farther [135]. Inhalation activities generally refer to inhalation from the nose and mouth, which affects the Inhalation doses of short- and long-range contaminated air. The parameters associated with virus-particle inhalation include the inhalation rate and respiration frequency, which increase with activity level (Table 14). It is estimated that people spend, on average, less than an hour a day in moderate and high levels of activity [136]. Gender and age also play a role, compared with adult females, adult males have higher inhalation rates and lower respiratory frequency.

**Figure 20:**
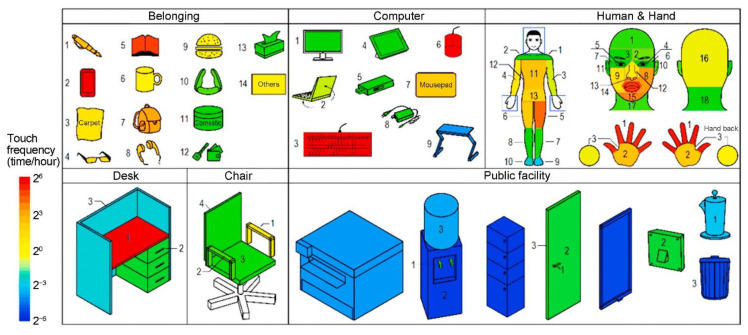
Touch frequency for six categories of surfaces in the student office [130].

Human-object interaction mainly involves touching inanimate surfaces, during which the virus is transferred between hands and surfaces. It is generally considered that the contact area of a finger is 1 cm^2^. Zhang et al. [72] investigated surface touching in a graduate office (Figure 20) and found that the average touch frequency of students was 5/min, and each time lasted 22 s on average, meaning that the students touched during 94.6% of the observation period. The majority (68%) of people have been exposed to public surfaces, but for individuals, public surfaces only account for 1.2%. Water cooler buttons and printers were touched by an average of 15 and 6.4 students per day, with individuals touching each time for an average of 10 s, but with a frequency of less than once per hour. The hand consists of three parts: the fingers, the palm and the back of the hand to adapt to different touch actions (Figure 20), and their touch frequencies are 479/h, 327/h and 51/h, respectively [130]. The right-hand touches more frequently than the left [130], as the right hand is the dominant hand for most people. 

Self-inoculation refers to the transfer of the virus by touching the facial membrane of susceptible individuals with contaminated hands (Figure 20), which is related to personal habits. Zhang et al. [130] observed that students touched their facial membrane on average 61.3 times per hour, and their faces more than 10 times per hour [72]. Hendley et al. [137] surveyed 124 adult subjects and found that only one of every three subjects picked his nose, and one of every 2.7 rubbed his eye in one hour. The combined rate of nose-picking and eye-rubbing episodes has been reported to be 0.7/h [137]. Another observation of 30 people showed that the average frequency of hand contact with the eyes, nostrils and lips was 15/h [138]. Note that fingers account for 90.2% of touches (mostly from the non-dominant hand) and palms account for 7.6% [130]. Detailed self-vaccination data helps calculate the risk of surface-based transmission.

Micro-pedestrian models, such as social-force models, cellular automata models, and agent-based models, have been used to simulate individual context-related behavior and close human-to-human proximity to assess the effectiveness of policies regarding physical distancing [139]. However, few studies on infection risk involve simulations of touching fomites, due to the lower risk of fomite transmission compared to airborne transmission.

### 5.3. Virus Spread Model

The simulation of the spread of the virus is closely related to the movement status of the occupants. For droplet transmission and airborne transmission, some studies assume that all occupants remain stationary, and according to different research objectives, there are generally two considerations: (1) calculate the path of each droplet to restore the real conditions, and (2) assume that the droplets are uniformly distributed in the space immediately after being exhaled to simplify the calculation. The CFD technique allows for the tracking of detailed trajectories of droplets from being exhaled to landing or being suspended in the air and is widely applied in modeling the transport and distribution of virus particles [140]. The Lagrangian approach and Eulerian approach are widely used, but their computational process is complex and time-consuming [140]. Other studies simplify droplet concentration distributions as a function of distance and combine it with individual movement behavior. It is generally assumed that infection may occur only within a certain distance (such as 1 m), so it is not suitable for aerosol transmission. Physical factors involved in a surface-based spread include touch force, friction, surface roughness, etc. [100]. Friction was shown to be effective in increasing the transfer rate of microorganisms and, in this case, the transfer rate was positively and negatively correlated with touch force and surface roughness, respectively [100]. In the absence of friction, increasing surface roughness still reduces the transfer rate, while touch force has no significant effect on transfer rate [100].

### 5.4. Infection Risk Calculation Model

Calculating the risk of infection involves estimating the dosage received by an individual and then calculating the probability of infection under a given dose. The dose-response model allows for a more complete and accurate assessment of infection risk from the multi-route transmission, including droplet, airborne and contact transmission. When the distribution of the virus in the accessible medium follows the Poisson probability distribution, the dose-response model can be expressed as [122]:

(1) $$P_{I}=\sum_{k=1}^{\infty }\frac{{\left(rN\right)}^{k}\exp\left(-rN\right)}{k!}$$

where *P_I_* (%) represents the infection probability, *k* is the number of virus species ingested and surviving in the host, *r* is the probability of a virus surviving in the body and causing an infection and *N* is the intake dose. This dose-response equation becomes an exponential equation through the simplification of the summation series:

(2) $$P_{I}=1-\exp\left(-rN\right)$$

It reflects the interaction between the human body and the virus and the infectivity of the virus. When there are multiple transmission routes and the infectivity of the virus varies with the deposition site, the total infection risk through multi-route transmission can be calculated by Equation (3):

(3) $$P_{I}=1-\exp\left(-\left(\theta_{t}D_{dc}+\theta_{t}D_{pf}+\theta_{d}D_{ld}+\theta_{i}D_{sa}+\theta_{t}D_{ef}+\theta_{i}D_{la}\right)\right)$$

where *D_dc_*, *D_pf_*, *D_ld_*, *D_sa_*, *D_ef_*, *D_la_* are cumulative exposure doses due to direct contact transmission, personal fomite transmission, large droplet transmission, short-range airborne transmission, environmental fomite transmission and long-range airborne transmission, respectively. *θ_t_*, *θ_d_*, *θ_i_* are the dose effects of touching the facial membranes, deposition in the upper respiratory tract, and direct inhalation, respectively.

The detailed spatial and temporal distributions of virus concentrations in the environment can help to obtain the intake dose for each transmission route. According to the proposed framework, the key issue for the co-simulation of 3D building models, occupant-behavior models and virus-diffusion models is the interaction of individual movement behavior data with CFD simulation data of droplet and aerosol diffusion. Yu et al. [140] transformed the steady-state airflow velocity field in the CFD model into a cellular automaton model to calculate the indoor pollution particle concentration, which improved the efficiency by about five times while ensuring accuracy.

There are no studies yet to accomplish the ideal interaction of space, occupants and virus particles in one environment so far as we know. The agent-based model (ABM) can simulate the complex motion of multiple agents, and the bidirectional coupling with CFD has been realized in the field of biology. The occupants and viruses are regarded as dynamic agents, and the 3D architectural space is regarded as the activity environment of the agents. ABM can effectively describe the behavioral characteristics of environmental elements and agents and their micro-interactions, which provides an opportunity to couple the building-space model, the occupant-behavior model and the virus-transmission model.

### 5.5. Case Study

**Figure 21:**
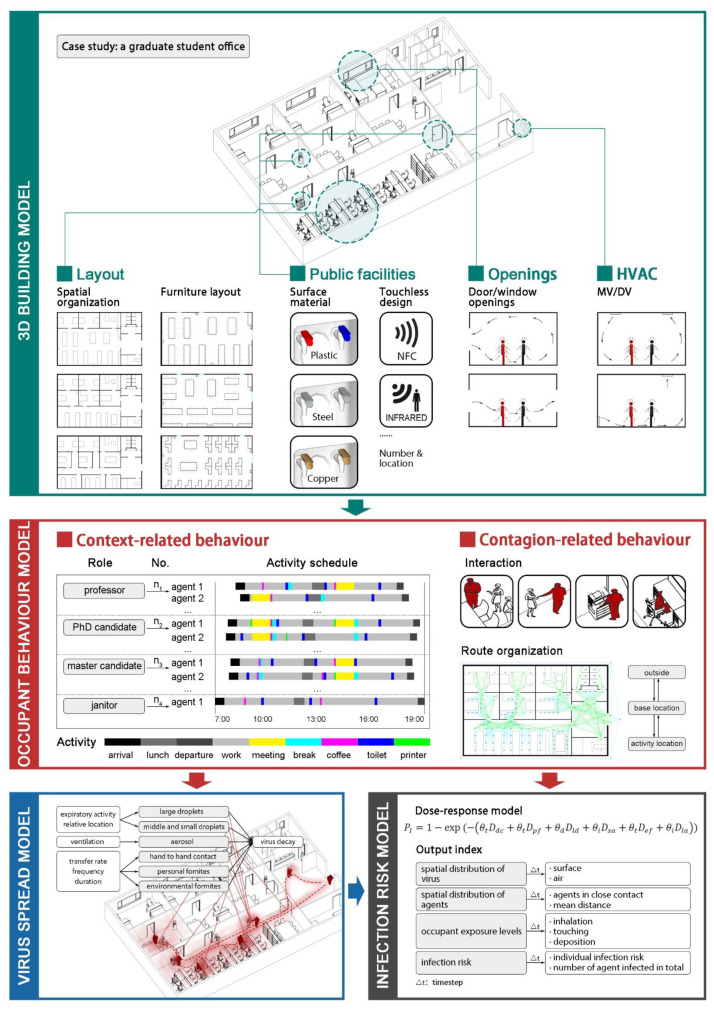
The graduate office building case illustrating the space-based framework.

A hypothetical graduate student office is used as an example to illustrate the workflow of the framework (Figure 21), with a relatively simple floor plan and organizational structure. The building contains an entrance, toilet, guard room, office space, meeting space and rest space, with an area of 380.8 square meters. 

For the 3D building model, it is assumed that there are three forms of spatial organization: open, semi-open and closed and, in each case, there are three furniture layouts. Then a 3 × 3 layout matrix is generated. Moreover, the door handles, water dispenser switches and printer buttons in buildings are considered frequently touched surfaces. There are three optional materials for each surface, resulting in a 3 × 3 × 3 material matrix. A facility will not be considered in the environmental-contamination level calculation if its property is “touchless”. In addition, since most rooms in the building cannot be naturally ventilated, two types of mechanical ventilation (mixed ventilation (MV) and displacement ventilation (DV)) were chosen. Finally, a (3 × 3) × (3 × 3 × 3) × 2 matrix containing types of spatial factors is generated.

For the occupant-behavior model, there are four roles in the office (professor, doctoral candidate, master candidate, and janitor), each with n_1_, n_2_, n_3_, and n_4_ agents, respectively. There are nine potential activities for each occupant during the day: arrival, lunch, departure, working, meeting, breaking, drinking coffee, going to the toilet, and printing, of which the last six activities can be considered as states of the Markov chain. Each role is assigned a basic activity schedule, based on which everyone makes minor adjustments to fit his habits. The route organization is generated by combining occupant-activity information with building information. The shortest path algorithm is selected assuming everyone is familiar with the office. At this stage, parameters such as the number of close contacts, average interpersonal distance, etc., will be used as indicators of high-risk behaviors in the space.

For the virus-spread model, a random agent is selected as an infected individual, and the spread of the released virus can be calculated according to his/her activity network. The result is greatly affected by the role of the infected individual. The total exposure of the susceptible is the sum of the exposure from the air-based route and the surface-based route.

For the infection risk prediction model, the dose-response model can be used to calculate the infection risk of a susceptible individual exposed by different transmission routes, and further predict the total infected population during building operation. In a complete workflow, the input parameters include the spatial factors of the office building, the occupant behavior features and the virus-spread data, and the output results consist of the spatial distribution of virus and agents, the occupant-exposure levels and the infection risk, etc.

## 6. Discussion and Limitations

Given that most COVID-19 infections occur indoors, the whole process of RDs from release to ingestion was summarized in this paper, and the influencing factors involved were divided into three categories: space, environment, and behavior. This study highlights the role of spatial factors in individual infection-risk assessment, which include three aspects: spatial layout, public-facility design and opening design. They affected droplet-based, surface-based, and air-based transmission of the virus by intervening in occupant behavior and indoor airflow fields, respectively. In addition, changes in surface material and environmental factors (AT, RH, and daylighting) caused by opening design also have an impact on the virus viability.

Most of the current research on the impact of spatial layout on occupant behavior is observational analysis, and the combined effect of multiple variables is difficult to predict. For example, a one-way space layout may not necessarily help reduce exposure risk, as the number of customers in a specific space decreases, their exposure time may increase. Spatial configuration and physical layout are complex issues in architectural design, especially for large public buildings. The detailed analysis of specific cases using space syntax can help to examine the effectiveness of spatial layout in reducing infection risk. Functional structure, circulation patterns, and horizontal and vertical accessibility are important factors affecting occupant distance and duration. Although the diversification of space functions is beneficial to space utilization, it is easy to cause the mixing of different groups and increase the probability of cross-infection. The multi-functional space is only allowed to undertake one function in a period through reasonable planning and management to avoid people from gathering at the same time. Enhanced spatial connectivity results in increased viral-diffusion efficiency, including the diffusion of contaminating gases and the spread of viruses on surfaces. The clearly structured circulation-pattern facilitates wayfinding and saves people unnecessary gathering time. While human-human and human-object interactions are primarily based on social relationships and schedules, their frequency and location preferences are still influenced by spatial configuration. Good visual connectivity increases unplanned interactions, such as open-plan offices for enhanced employee communication. Overall, it is easier to predict the impact of spatial factors on user movement and distance than the impact on interaction behavior.

COVID-19 has helped us understand the importance of touchless infrastructure, but its contribution to reducing the risk of infection has not been quantified. Furthermore, the design and implementation of touchless products have two limitations: high economic cost and restricted accessibility for people with disabilities. But it is indisputable that touchless design will play an important role in building epidemic prevention and driving the development of smart buildings.

The configuration of door and window openings includes location, size, and type, and its effect on the diffusion of contaminated air has generally been underestimated in previous studies. The effects of ventilation modes and ventilation rates on infection risk have been widely discussed over the past few years. José Luis Jiménez and his team evaluated parameters governing ventilation inside buildings [141] and vehicles [142], and proposed effective actions. Allen et al. [143] suggested that increasing air changes per hour and air filtration is a simplified but important concept that could be deployed to help reduce risk from within-room, far-field airborne transmission of SARS-CoV-2. Furthermore, different Spanish research groups have developed a Aireamos [144] platform employing various types of ventilation strategies by analyzing CO_2_ levels to assess and control the indoor COVID-19 infection risk. In addition, Henriques et al. [145] have established a COVID-19 airborne risk-assessment methodology, which proved that single-sided, naturally ventilated classrooms with windows slightly open in winter can result in a 2.2-fold reduction in the infection dose. In addition to ventilation efficiency, the location of the infected individual also needs to be emphasized. If the infected person is around the upper air vent, the better the ventilation, and the faster the spread of the contaminated aerosol.

Changes in one space-design parameter may cause multiple reactions, such as the opening area of windows not only affects the diffusion of polluting gases and the virus viability, but also affects the thermal comfort of occupants and the energy consumption of buildings. The current research on building optimization focuses on comfort and energy efficiency, and standards for ventilation, temperature and humidity in public spaces also address indoor air quality and thermal comfort. The anti-epidemic performance of buildings is emphasized in this paper. For example, setting a minimum RH standard of 40% for public buildings will not only reduce the impact of COVID-19, but will also reduce the impact of further viral outbreaks, both seasonal and novel [146]. Guidelines may also need to be improved to meet risk-control requirements during a pandemic and address the concerns of building practitioners.

To calculate the comprehensive infection risk of indoor COVID-19, six possible transmission routes, as well as the spatial, environmental and behavioral factors involved are summarized. A spatially sensitive, multi-route risk-assessment framework is then proposed in this paper. The proposed framework helps building practitioners and managers to gain an overall perception of the risk of contagion in different building spaces, as well as the transmission routes that contribute the most, to propose targeted interventions. The framework required (1) to be able to control the detailed spatial and environmental parameters, (2) high-fidelity simulation of occupant context-related behavior and contagion-related behavior, (3) coupling the microscopic behavior model with the virus spread model and, (4) allowing for efficient computation and visualization.

This work emphasizes the importance of complex space-human-virus interactions, of which three key issues are involved: (1) First, human-space interactions require detailed data to ensure high fidelity and randomness of behavioral simulations, which are different from those of single, generalized motion patterns. Microscopic behavior simulation has been extensively studied in the field of building safety and energy consumption. Context-related beha vioral data can be obtained experimentally (wireless location systems, video recordings, self-reports, etc.); yet, traditional observation and self-reporting fail to meet the accuracy and precision required for infection-related behaviors, especially respiratory activities and touch behaviors. Due to privacy and technological constraints, there is little data on occupant exposure habits. Only the surface touch network of office workers has been studied, but specific details such as contact area and friction remain unclear. (2) Second, space-virus interaction is related to the virus spread and survival, which has usually been ignored in previous studies. While 70% of virus particles in the air will be deposited on environmental surfaces, the spread of the virus to all surfaces in the space, this is rarely considered in the study of contact transmission. Most conclusions about the impact of surface material, ambient temperature and humidity and sunlight on the virus viability are based on laboratory measurements. More research is needed to obtain the viability of the virus in the real environment, which can be disturbed by a variety of factors. (3) Third, the dynamic interaction between space, humans and viruses requires coupling the microscopic behavior model and the virus-spread model and adding the obtained data to the infection-risk calculation model. Most of the current research only focuses on a single airborne or contact transmission. The co-simulation of CFD and ABM provides an opportunity to calculate the exposure dose of the virus for multi-path transmission. In addition, one of the other limitations in the COVID-19 infection risk assessment is the large inconsistency of the SARS-CoV-2-related virus data in calculating the exposure doses and infection risk. There are different opinions about the virus mortality rate, the size and number distribution of exhaled droplets, the viral load in the droplets, and the parameters in the dose-response formula.

Our findings highlight the potential contribution of building-space design in the control of the spread of respiratory infectious diseases. The effectiveness of nonpharmacological interventions, such as increasing the air-change rate and the frequency of hand washing, and social distancing, was widely discussed. However, the effectiveness of possible spatial interventions has not been emphasized. The impact of single spatial measures requires further research, as well as the combined effect of multiple interventions. 

Effective control measures for a specific space should be proposed based on the characteristics of the occupants and their activities. As presented in the case study of the graduate student office, most agents work up to 8 h and their location changes frequently with roles and activities. Potentially effective interventions for the building include: (1) Spatial measures need to be implemented according to on-site ventilation conditions, population density, occupant behavior and spatial conditions. For example, dividing the office area into smaller units to avoid excessive crossing of routes and large-scale aerosol diffusion could be an effective measure under normal- or high- efficiency ventilation conditions [80,94]. However, with poor ventilation, aerosols in cubicles cannot be diluted by clean air and are more likely to accumulate in the room, leading to high infection risk of occupants. (2) Aligning desks and chairs in the same direction to reduce face-to-face contact [83,95]. (3) Preferentially using infrared sensing technology at the entrance of office areas and meeting rooms [72,89]. (4) Considering antimicrobial materials for water dispenser switches and printer buttons in public office areas, and cleaning frequently [72]. All these architectural interventions can help architects and building managers to make better decisions to reduce indoor infection risk. Note that the effectiveness of interventions depends largely on the relative contributions and efficiencies of the various transmission routes. This uncertainty stems from the interaction of pathogens, occupants and space. Therefore, interventions should be proposed and implemented in consideration of all three aspects of the framework.

One of the limitations is that although multiple transmission routes are considered in this article, there are still some potential or actual transmission routes not covered by this article, which, to our current knowledge, lack of sufficient evidence from cases and academic literature, such as the faecal-oral transmission and the vertical spread of virus-laden aerosols through stacks and vents. The lack of evidence is due to the fact that the detection and tracking of virus particles is limited by venues and technologies, and the sampling and handling of virus requires specialized and safety laboratories, which are not available to general researchers. Moreover, in real scenarios, multiple transmission routes appear in combination and cannot be completely distinguished. Once infection occurs, even if there is evidence of viral transmission, especially via the aerosol route, the spatial impact of individual infection is not easily demonstrated.

## 7. Conclusions

This study reviews the multi-route transmission of SARS-CoV-2 in indoor spaces and investigates the spatial, environmental, and behavioral factors involved and their relationships. The spatial factors affecting the indoor virus spread include three aspects: spatial layout, public-facility design and opening design. This paper summarized the role of spatial factors during virus transmission, including affecting the movement of the virus by intervening in occupant behavior and indoor-airflow field and affecting the virus viability by changing the surface material and environmental conditions etc. Furthermore, a space-based, multi-route infection-risk assessment framework is proposed, aiming to build a big picture of indoor infection risk assessment, while helping to understand and analyse the effectiveness of different spatial, environmental and behavioral interventions more accurately. A graduate student office is used to explore the possibility of applying the framework. Our future work will focus on the data collection required in this framework to enable co-simulation of complex interactions between space, occupants, and viruses. 

## Figures and Tables

**Table 1:** The initial characteristics of droplet produced by different expiratory activities.

Expiratory Activity	Size Range (μm)	Emission Rate ^a^	Particle Concentration (L^−1^)	Velocity (m/s)	Duration per Time (s)
Breathing ^b^	0.01–100 [34]	0–65 [35] 85–691 [32]	1–13 [36] 100–450 [37] 18–890 [33]	<1.4 [38] 0.45–2.58 [39]	3.3–5 [40]
Speaking ^c^	<0.1–125 [34] 0–1000 [16,45] 5–2000 [35]	50–770 [35] 120–1380 [32] 1–374/s [16] 176 [46] 112–6702 [45]	6–36 [36] 3–50 [35] 4–223 [45] 16–370 [33] 150–2000 [37]	2.31–4.07 [40] 3.9 [41] <6.25 [44]	-
Coughing	<0.1–500 [34] 0–1000 [45] 0–1500 [16] 5–2000 [35]	465 [46] 271–1331 [16] 947–2085 [45] 490–16,000 [35]	24–218 [36] 6–910 [35] 150–2500 [37] 2400–5200 [45] 220–41000 [33]	1.5–28.8 [45] <4.5 [40] 11.7 [41] <15.3 [42]	0.3–0.8 [40]
Sneezing	<0.1–125 [34] 5–2000 [35]	65,000–3,100,000 [35]	5–73,000 [35]	<4.5 [40] 30 [43] <15.9 [44]	0.15–0.25 [40]

^a^ The emission rate for breathing, speaking and singing is the number of droplets per second. The emission rate for sneezing and coughing is the number of droplets each sneezing and coughing. ^b^ Mouth breathing is not included. ^c^ Normal volume of 50–60 dBA.

**Table 2:** Recommended classification of transmission routes.

	Short-Range Exposure				Long-Range Exposure	
Transmission route	① Direct contact route	② Personal fomite route	③ Large droplet route	④ Short-range airborne route	⑤ Environmental fomite route	⑥ Long-range airborne route
Exposure way	Touching	Touching	Deposition	Inhalation	Touching	Inhalation
Virus vector	Body fluid and skin	Personal fomites	Large droplets	Middle and fine droplets, droplet nuclei	Environmental fomites	Fine droplets and droplet nuclei
Propagation distance	0 m	≤1 m	≤1 m	≤1.5 m	>1.5 m	>1.5 m

**Table 3:** Distribution of SARS-CoV-2 on surfaces in different spaces (No. of RNA-positive samples/total No. of samples (%)).

Sites	Spaces	Surfaces		
		Envelopes ^a^	Public Facilities ^b^	Personal Belongings ^c^
Total	130/674 (19.3)	1/18 (5.6)	105/457 (23.0)	24/199 (12.1)
Hospital [69]	Total—84/616 (13.8)			
	ICU—22/69 (31.9) Obstetric isolation ward—9/32 (28.1) Isolation ward—11/56 (19.6) Outpatient lobby—5/30 (16.7) Emergency department—10/80 (12.5) Office and preparation area—5/41 (12.2) Obstetric ward—4/33 (12.1) Clinical laboratories—11/96 (11.5) Fever clinic—3/46 (6.5) CT examination room—2/36 (5.6) General ward—3/55 (5.5)	Wall and floor—1/18 (5.6)	Door handle—12/75 (16.0) Hand-sanitizer dispensers—12/59 (20.3) Self-service printer—2/10 (20.0) Table top/keyboard—29/173 (16.8) Medical equipment—6/48 (12.5) Elevator buttons, microwave ovens, faucets, handrails, and hair drier—2/25 (8.0)	Gloves—12/78 (15.4) Telephone—7/56 (12.5) Eye protection or face shield—1/58 (1.7)
Bank [70]	Total—4/9 (44.4)	-	Door handle—3/6 (50.0) ATM—1/3 (33.3)	-
Shop [70]	Total—21/27 (77.8)	-	Door handle—19/24 (79.2) Basket handle—2/3 (66.7)	-
Restaurant [70]	Total—5/15 (33.3)		Door handle—1/3 (33.3) Trash can—4/12 (33.3)	-
Quarantine hotel [71]	Total—16/42 (38.1)			
	Hotel room—7/21 (33.3) Public space—9/21 (42.9)	-	Toilet seat—1/1 (100.0) Electric kettle—2/2 (100) Cold water bar—1/1 (100.0) Air sampling filter—1/1 (100.0) Elevator button panel—4/5 (80.0) Closet door—1/2 (50.0) Chair handle—2/4 (50.0)	Cup—3/3 (100.0) Telephone—1/4 (25.0)

^a^ Include ceiling, wall, and floor. ^b^ Include window and door handle, ventilator, light switch, elevator button, furniture (chair/desk/bed/locker et al.), sanitary appliance (sink, faucets, toilet seat, toilet bowl et al.), electric appliance (printer, microwave ovens, hair drier et al.). ^c^ Include telephone, shoes, gloves, walker, eyeglasses, cup, mouse, and keyboard.

**Table 4:** Differences of virus exposure between various spaces (taking school as an example).

Space	Classroom	Office	Dormitory
**Schematic diagram**	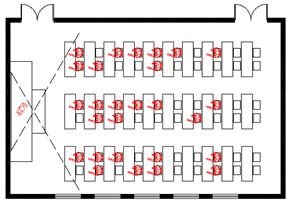	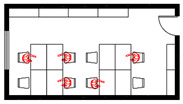	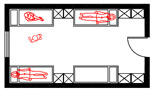
**Occupant role**	Student, teacher	Teacher, administrator	Student
**Activity (level)**	Attending class (light)	Working, talking (light)	Rest, sleep (resting)
**Motion state**	Stationary	Stationary	Stationary
**Duration (min)**	About 40/lesson	About 40/lesson	>420
**Possible contagion- related behavior**	Teacher speaking; close contact between students; touching desk, chair, door handle; breathing	Talking to colleagues; touching desk, computer, door handle etc.; shaking hands; breathing	Talking and playing with roommates; touching door handle, chair, bed, wardrobe door, etc.; breathing
**Transmission routes**	R_pf_ R_ld_ R_sa_ R_ef_ R_la_	R_dc_ R_pf_ R_ld_ R_sa_ R_ef_ R_la_	R_dc_ R_pf_ R_ld_ R_sa_ R_ef_ R_la_
**Space**	**Stairwell**	**Corridor**	**lavatory**
**Schematic diagram**	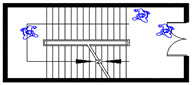	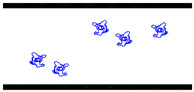	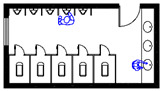
**Occupant role**	Student, teacher, sanitation worker, administrator	Student, teacher, sanitation worker, administrator	Student, teacher, sanitation worker, administrator
**Activity (level)**	Climbing stairs (moderate)	Walking (moderate)	Cleaning (light)
**Motion state**	Moving	Moving	Stationary
**Duration (min)**	<5	<5	<5
**Possible contagion- related behavior**	Talking to fellow travelers; crowd gathering; touching stair handrail; breathing	Talking to fellow travelers; crowd gathering; breathing	Touching flush button, faucet and door handle; breathing
**Transmission routes**	R_ld_ R_sa_ R_ef_ R_la_	R_ld_ R_sa_ R_la_	R_ef_ R_la_ R_fo_

R_dc_: direct contact route, R_pf_: personal fomite route, R_ld_: large droplet route, R_sa_: short-range air-borne route, R_ef_: environmental fomite route, R_la_: long-range airborne route, R_fo_: faecal-oral route.

**Table 5:** Description of spatial, environmental, and behavioral parameters.

	Parameter	Description	Symbol
Space	Layout	Floor-level spatial organization and room-level physical layout	S_L_
	Public facilities	Touchless technology and surface material	S_P_
	Openings	Door and window openings	S_O_
Environment	Airflow	The rate and direction of air flow, mainly dependent on ventilation	E_A_
	T and RH	Indoor temperature and relative humidity	E_TRH_
	Daylighting	Place windows, skylights, and other openings for sunlight	E_D_
Behavior	Human-air interaction	Respiratory activities, including breathing, speaking, singing, coughing, and sneezing	B_HA_
	Human-human interaction	Direct physical contact and close contact	B_HH_
	Human-object interaction	Touch inanimate surfaces, including personal belongings and public facilities	B_HO_
	Self-inoculation	Touching the facial membranes, including the eyes, nose and mouth with contaminated hands	B_S_

**Table 6:** Likert scale for the level of permeability and wayfinding [80].

Scale	Permeability	Wayfinding
Excellent	Hardly accessible by the public, and the private space is only dedicated to a specific and authorized person	The entrance visible without any obstruction by the user from the external area, and the internal space is easily accessed from the standing point
Average	A certain area still lacks in controlling the users, especially in segregating public and private users	Space can be accessed, however, depends on the depth of the space. The further it goes, the less quality of wayfinding it possesses
Poor	Easily accessible by public users without any space to pass through and weak in control of the movement of end-user especially undedicated areas	Weak in circulation and spatial organization as user-facing difficulties in accessing the space and it goes further from the main entrance

**Table 7:** Technologies, working range and use cases [90].

Technology	Working Range	Used Cases
Infrared	10 m	Doors, elevators, lights, water dispensers, toilet, sanitizing dispenser, faucets, hand dryers
Biometric authentication	0.5 m	Payments, doors, elevators, lights, ATM, smart homes
RFID	10 m	Payments, doors, elevators
NFC	10 cm	Payments, doors, elevators
QR code	Depend on the size of QR code	Payments, doors, printers

**Table 8:** The MFR for various WWR and AR (data retrieved from [92]).

**WWR**	0.01	0.0225	0.04	0.0625	0.09	0.125	0.16	0.2025	0.25
**MFR (kg/s)**	0.175	0.394	0.713	1.09	1.62	2.21	2.89	3.69	4.512
**AR**	0.36	0.56	0.64	1	1.562	1.75	2.78		
**MFR (kg/s)**	0.709	0.711	0.711	0.713	0.722	0.728	0.727		

**Table 9:** Ventilation effectiveness of three physical layouts [96].

Physical Layout		Layout A	Layout B	Layout C
Ventilation effectiveness	MV1	0.854	1.154	1.342
	MV2	0.736	1.258	1.173
	DV1	1.363	1.758	1.558
	DV2	1.061	2.698	1.661

MV1—mixed ventilation (inlet on the top and outlet on the bottom), MV2—mixed ventilation (inlet and outlet on the top), DV1—displacement ventilation (inlet and outlet on the same side), DV2—displacement ventilation (inlet and outlet on the opposite side).

**Table 10:** Persistence of SARS-CoV-2 on different types of inanimate surfaces.

Refs.	T (°C)/RH (%)	Copper	Stainless Steel	Glass	Plastic	Wood	Paper	Cloth	Vinyl
[97]	22/65		<7 d	<4 d	<7 d	<2 d	<3 h	<2 d	
[82]	21–23/65	<4 h	72 h < 4 d		72 h < 4 d		<24 h		
[99]	20/50		>28 d	>28 d			>28 d	<14 d	>28 d
[98]	22/40–50						>8 h	>4 h	

**Table 11:** Effects of AT and RH on the viability of SARS-CoV-2 on inanimate surfaces and in aerosols.

Refs.	Surface/Aerosol	T (°C)	RH (%)	Results	Correlation	
					T	RH
[99]	Stainless steel, paper note, polymer note, glass, cotton, vinyl	20, 30, 40	50	Increasing AT accelerates inactivation of SARS-CoV-2	−	
[71]	Plastic	22, 40, 50, 60, 70	50	Increasing AT accelerates inactivation of SARS-CoV-2	−	
[110]	Metal discs	4, RT, 30	30–40	SARS-CoV-2 decays slowly at all AT	NS	
[107]	polypropylene plastic	10, 22, 27	40, 65, 85	SARS-CoV-2 is more stable at low AT and extreme RH (40% and 85%)	−	
[108]	ABS plastic, stainless steel, nitrile rubber	24, 28, 35	20, 40, 60, 80	Increasing AT and RH accelerates inactivation of SARS-CoV-2	−	−
[109]	polypropylene	4, 21, 27	40, 85	SARS-CoV-2 is more stable at low AT and low RH	−	
[112]	aerosol of artificial saliva	19–22	40–60, 68–88	SARS-CoV-2 is more stable at higher RH		+
[112]	aerosol of tissue culture media	19–22	40–60, 68–88	SARS-CoV-2 is more stable at medium RH		−

+ Positive correlation, − negative correlation, NS no significant correlation.

**Table 12:** Four personal spaces.

Personal Spaces	Social Relations	Behaviors	Distance (m)
Intimate zone	Couple, parent-child	Kiss (lips, nose, forehead, face), hug, touch (body), whisper	0–0.45
Personal zone	Friends or family with positive interactions	Cheek kiss, hand kiss, hug, talk, party	0.45–1.2
Social zone	Colleagues, strangers	Greetings, bows, and handshakes	1.2–3.6
Public zone	Public figure	Speech, show	>3.6–7.6

**Table 13:** Relationship and seating choice. (Dots indicate preferred choices.)

	(a)	(b)	(c)	(d)	(e)	(f)
	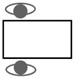	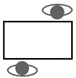	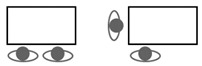		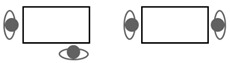	
**Talking**	●		●	●		
**Cooperation**	●			●		
**Collaborative**	●	●				
**Competition**	●	●				
**No interaction**		●			●	●

**Table 14:** Inhalation rate, frequency, and duration by activity level [136].

		Resting ^a^ (METS ≤ 1.5)	Light ^b^ (1.5 < METS ≤ 2.5)	Moderate ^c^ (2.5 < METS ≤ 5.0)	High ^d^ (METS > 5.0)
**Inhalation rate** **(m^3^/hour)**	Adult male	0.7	0.8	2.5	4.8
	Adult female	0.3	0.5	1.6	2.9
	Average adult	0.5	0.6	2.1	3.9
**Frequency (breaths/minute)**	Adult male	12	17	-	21
	Adult female	12	19	-	30
**Average hours per day ^e^ (hours/day)** **(percentage)**	Indoors	9.82 (40.91%)	9.82 (40.91%)	0.71 (2.96%)	0.1 (0.41%)
	Outdoors	0.51 (2.12%)	0.51 (2.12%)	0.65 (2.70%)	0.12 (0.50%)
	In vehicle	0.86 (3.58%)	0.86 (3.58%)	0.05 (0.20%)	0.0012 (0.01%)

^a^ Includes watching television, reading, and sleeping. ^b^ Includes most domestic work, attending to personal needs and care, hobbies, and conducting minor indoor repairs and home improvements. ^c^ Includes heavy indoor cleanup, performance of major indoor repairs and alterations, and climbing stairs. ^d^ Includes vigorous physical exercise and climbing stairs carrying a load. ^e^ Statistics for all age groups and genders.

## Data Availability

Not applicable.
